# Supplementary figures and images for: The Ca2+ concentration impacts the cytokine production of mouse and human lymphoid cells and the polarization of human macrophages in vitro
Source: PLoS One. 2023 Feb 24;18(2):e0282037. doi: 10.1371/journal.pone.0282037 (PMC9956017; doi:10.1371/journal.pone.0282037)

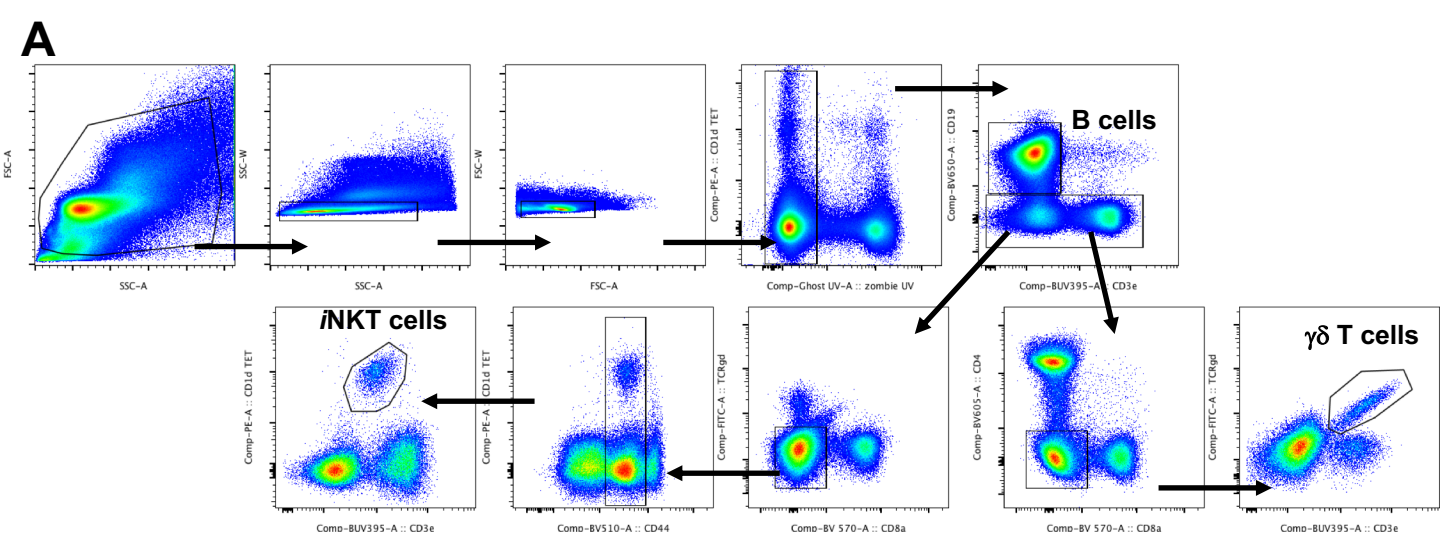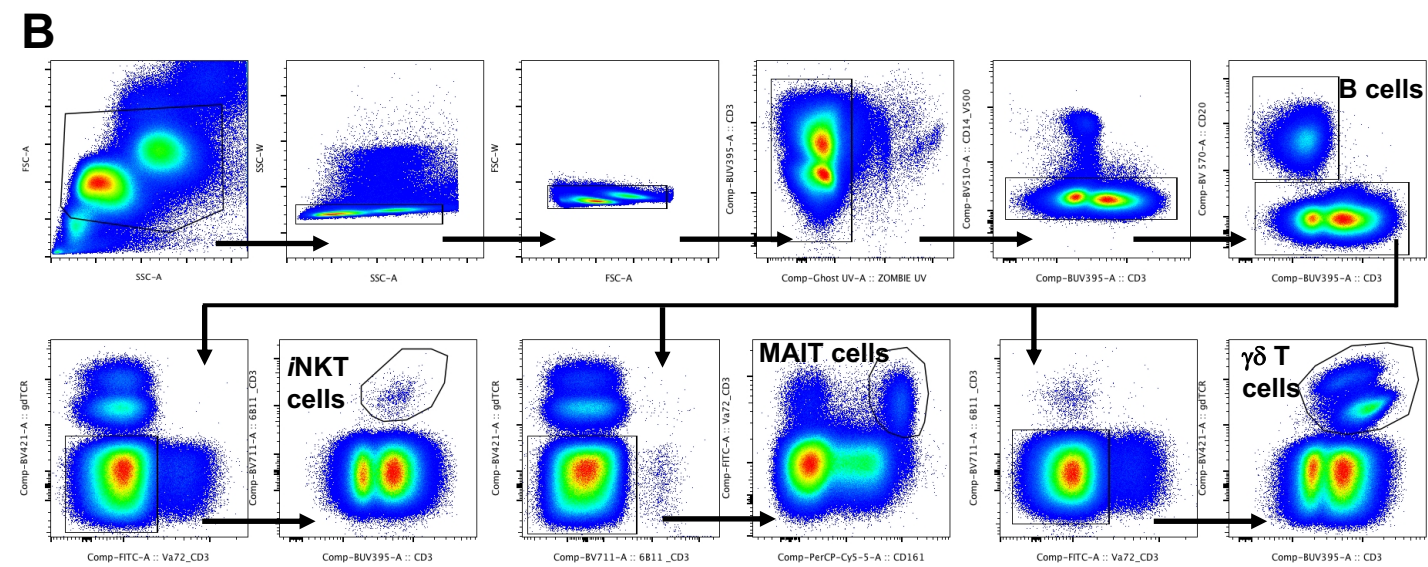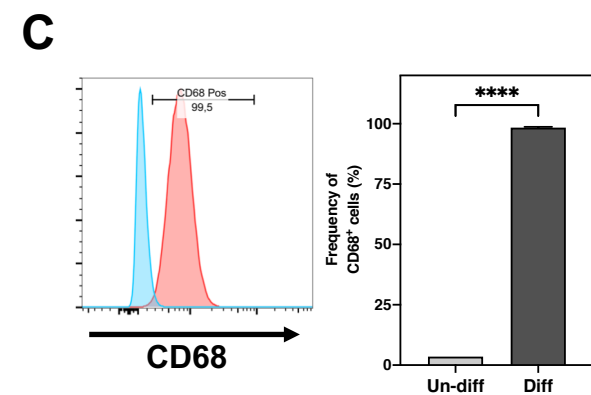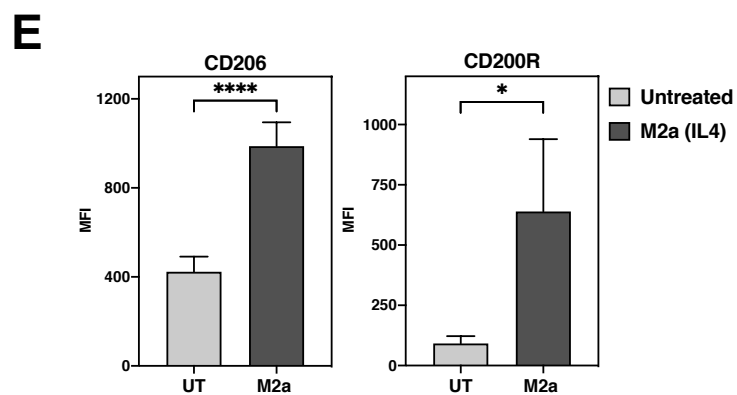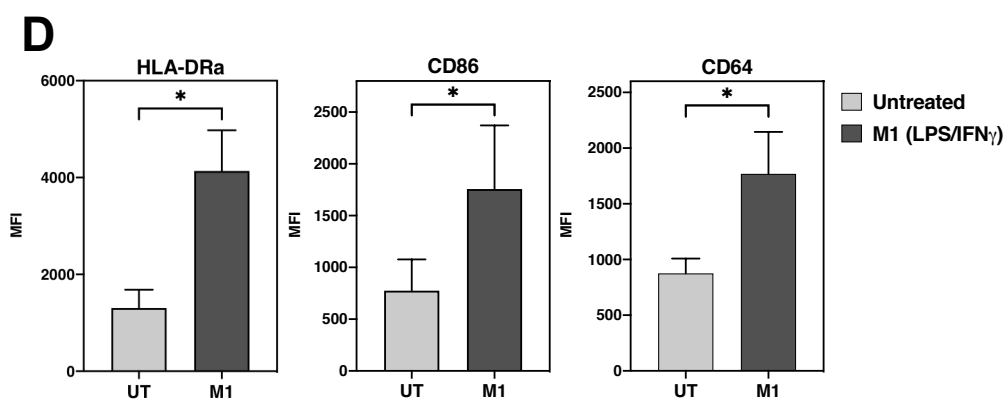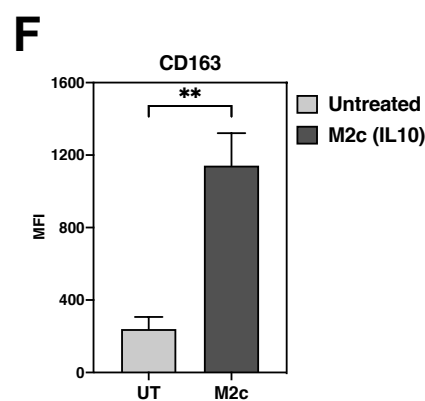

Supplement: S1 Fig — (A, B) Exemplary dot plots illustrating the gating strategy employed to identify the indicted (A) mouse or (B) human lymphoid cells. (C) Purity of the differentiated macrophages: human PBMC-derived monocytes were incubated for 7 days in RPMI1640 medium containing 10 ng/ml M-CSF for macrophage differentiation and the percentage of CD68+ macrophages was determined by intracellular staining. Left: representative flow cytometry data (blue = undifferentiated; red = differentiated); Right: Summary data (n = 3; un-diff = undifferentiated; diff = differentiated). (D-F) Evaluation of macrophage polarization. Primary human monocyte-derived macrophages were left unstimulated (UT) or stimulated for 12 h (D) with 100 ng/mL LPS and 20 ng/mL IFN-γ for M1 polarization (M1), (E) with 20 ng/mL IL-4 for M2a polarization (M2a), or (F) with 20 ng/ mL IL-10 for M2c polarization (M2c). Expression of indicated surface markers was analyzed by flow cytometry. The bar graphs indicate mean fluorescent intensity (MFI). The biological replicates of 6 independent donors pooled from 2 independent experiments are shown. (PDF) [file pone.0282037.s001.pdf]

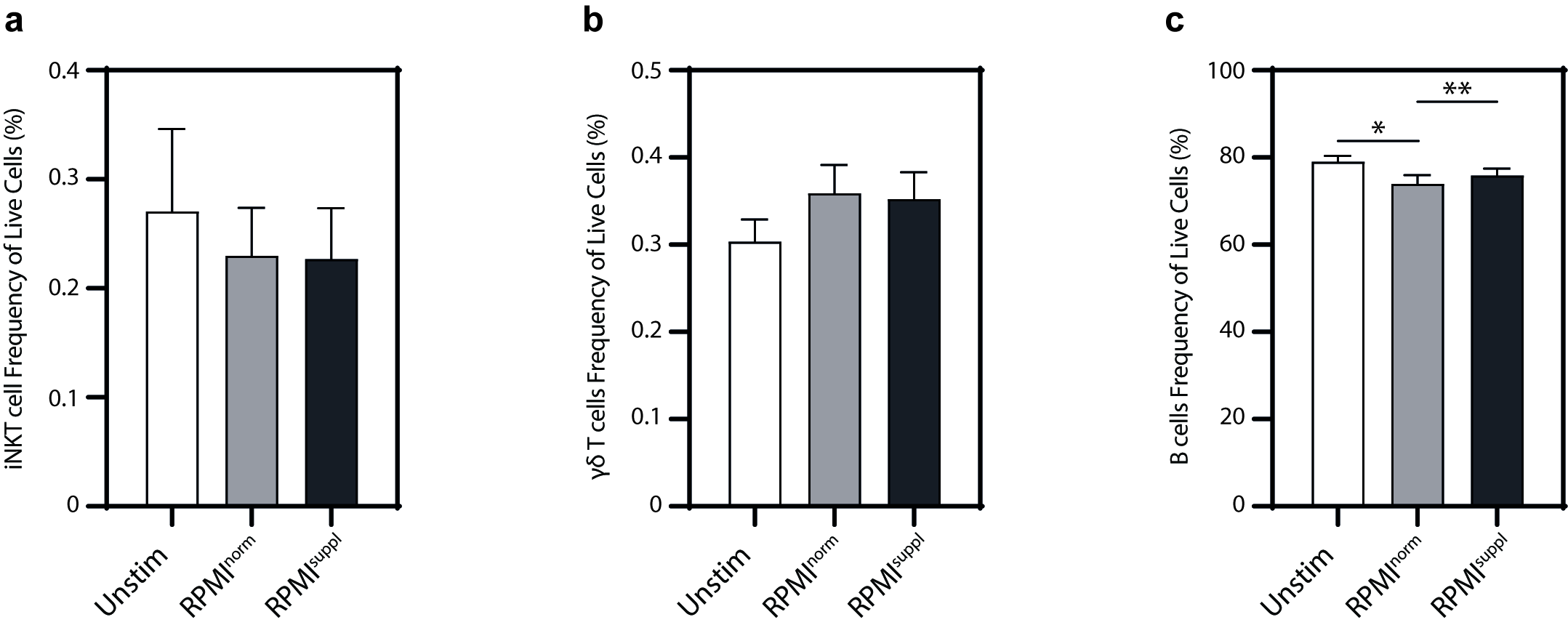

Supplement: S2 Fig — Splenocytes from C57BL/6 mice were stimulated 4 h with 50 ng/ml PMA and 1 μg/ml ionomycin in either normal RPMI1640 medium (RPMInorm) or RPMI1640 medium supplemented with 1 mM Ca2+ (RPMIsuppl). (a) iNKT cells; (b) Vδ2+ T cells; and (c) B cells were stained and analysed by flow cytometry. The bar graphs show the relative percentages of cells positive for LIVE/DEAD Fixable Blue Dead Cell Stain, indicating dead cells. Data were pooled from three independent experiments with three mice per group per experiment (n = 9). (TIF) [file pone.0282037.s002.tif]

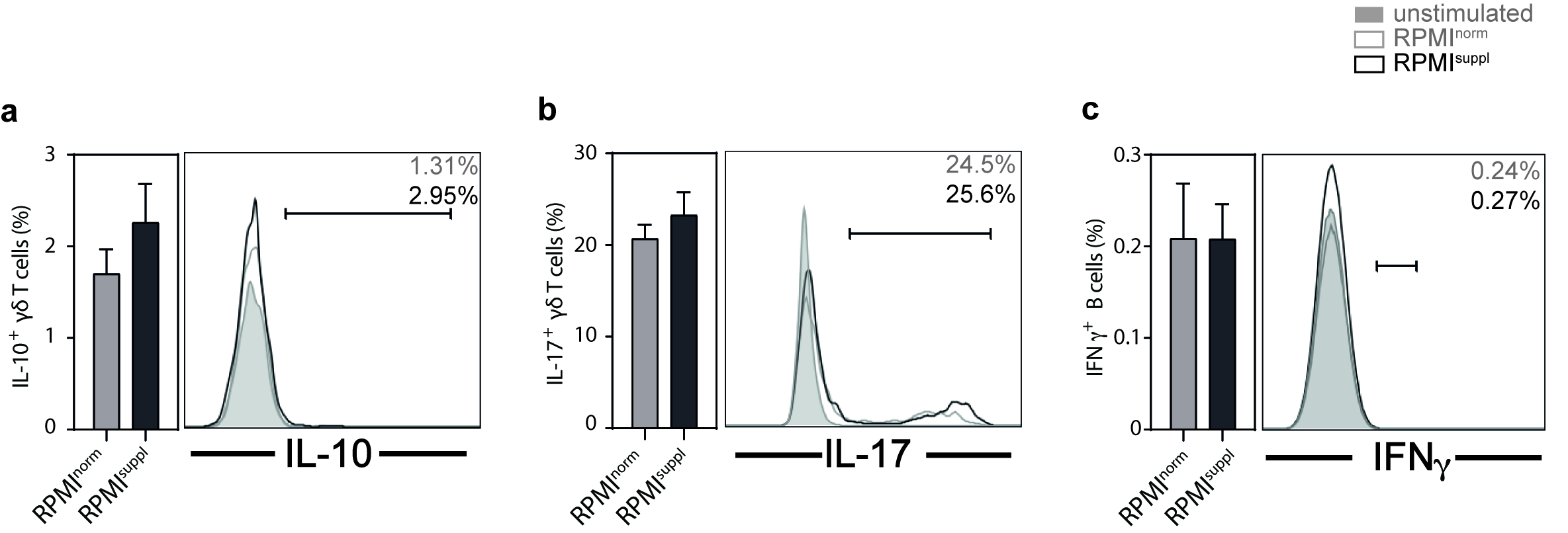

Supplement: S3 Fig — (a-c) Splenocytes from C57BL/6 mice were stimulated 4 h with 50 ng/ml PMA and 1 μg/ml ionomycin in either normal RPMI1640 medium (RPMInorm) or RPMI1640 medium supplemented with 1 mM Ca2+ (RPMIsuppl). The production of (a) IL-10 and (b) IL-17A by γδ T cells (live CD19/CD45R- CD4- CD8α- CD3ε+ γδTCR+ cells) and the production of (c) IFNγ by B cells (live CD3ε- CD4- CD8α- CD19/CD45R+ cells) was analysed by ICCS. Summary graphs (left panels) and representative data (right panels) from gated γδ T cells and B cells are shown, respectively. Data were pooled from three independent experiments with three mice per group per experiment (n = 9). (TIF) [file pone.0282037.s003.tif]

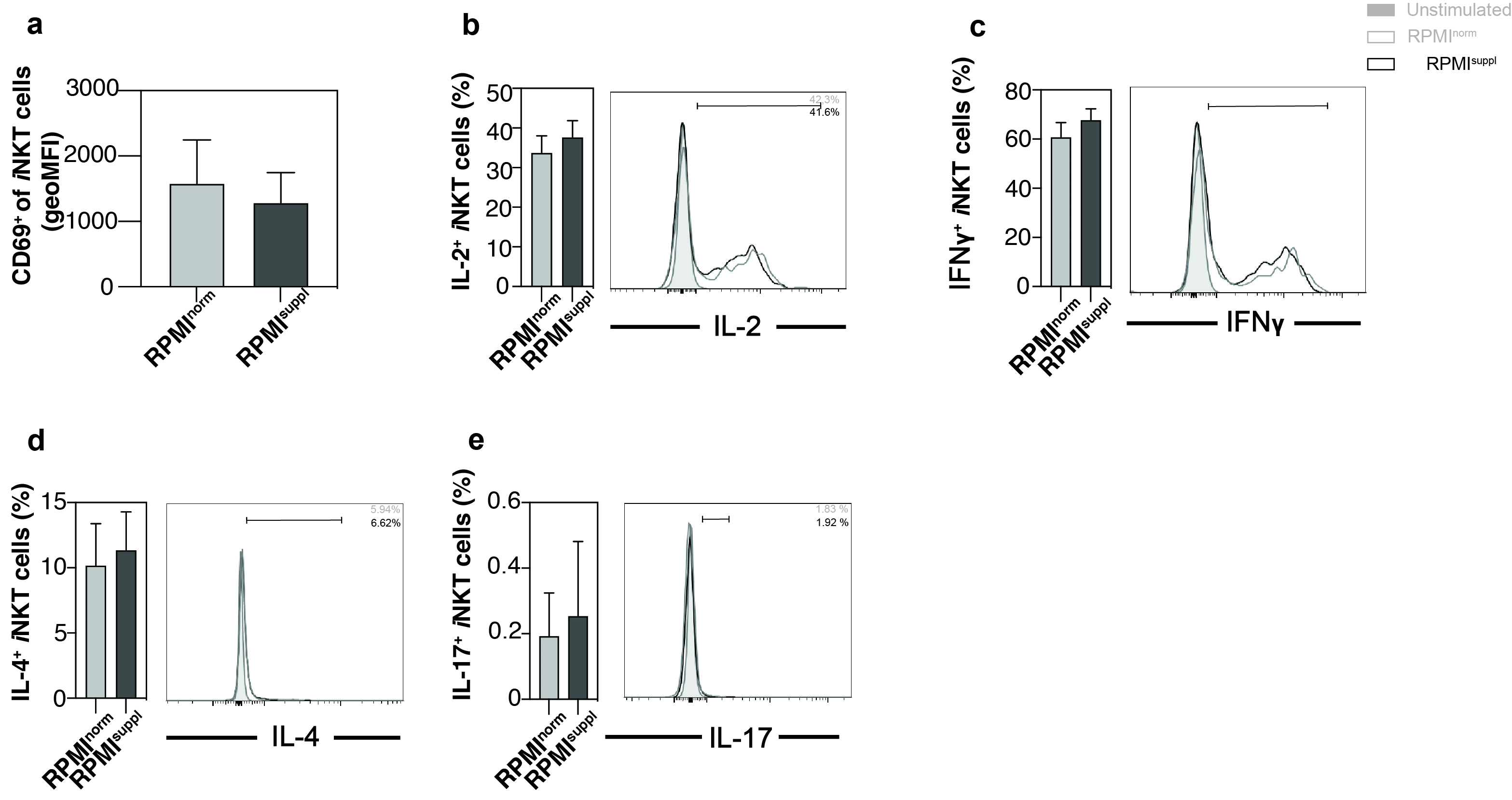

Supplement: S4 Fig — PBMCs were isolated from the residual leukocyte units of healthy donors. PBMCs were stimulated for 4 h with 25 ng/ml PMA and 1 μg/ml ionomycin in either normal RPMI1640 medium (RPMInorm) or RPMI1640 medium supplemented with 1 mM Ca2+ (RPMIsuppl). Human Vα24i NKT cells (live CD14- CD20- CD3+ 6B11+ cells) were analysed for the expression of the activation marker (a) CD69 and the production of the cytokines (b) IL-2, (c) IFNγ, (d) IL-4, and (e) IL-17. Summary graphs (left panels) and representative data (right panels) from gated iNKT cells are shown, respectively. Data were pooled from three independent experiments with three samples each (n = 9). (JPG) [file pone.0282037.s004.jpg]

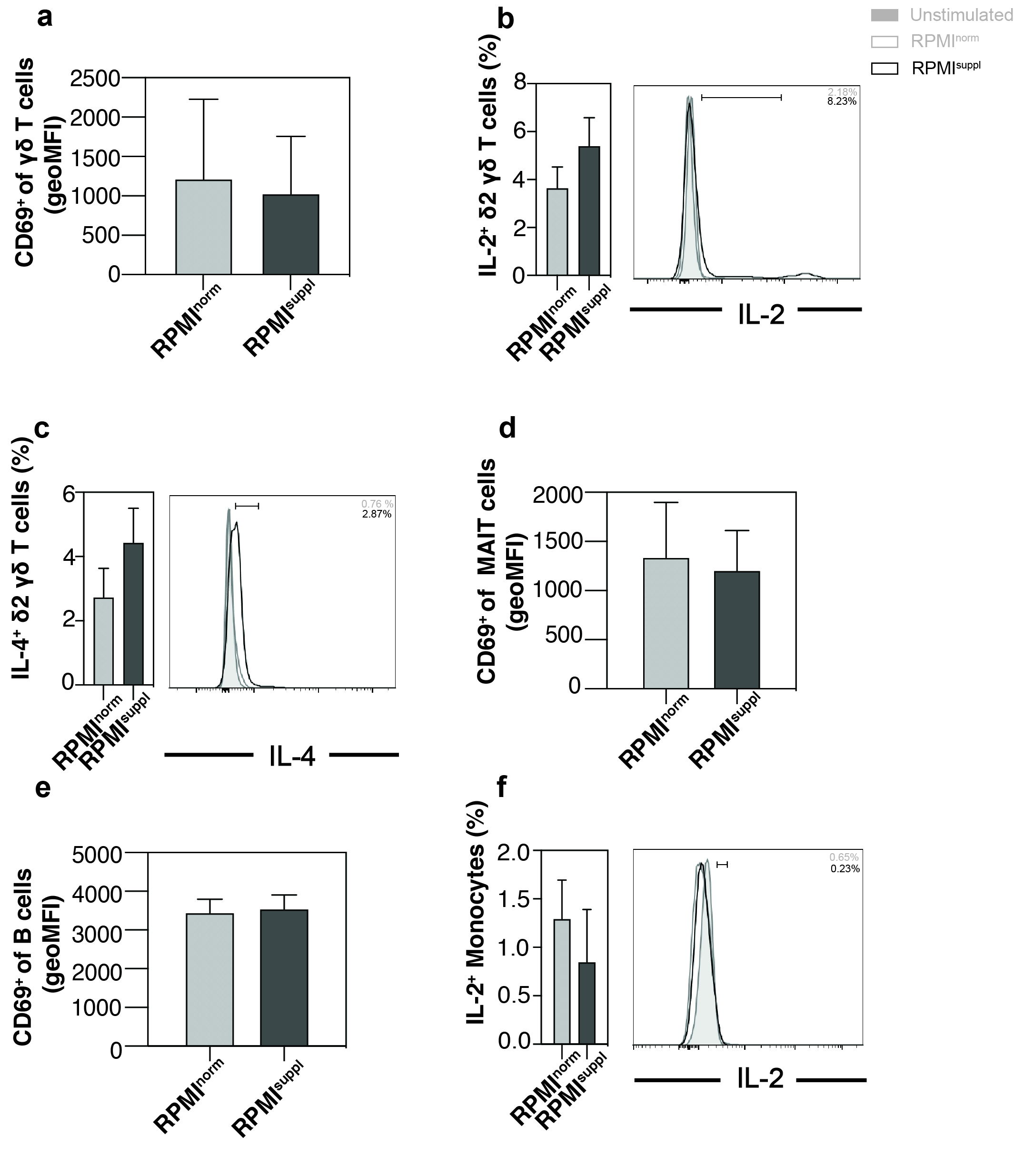

Supplement: S5 Fig — PBMCs were isolated from residual leukocyte units of healthy donors and were stimulated for 4 h with 25 ng/ml PMA and 1 μg/ml ionomycin in either normal RPMI1640 medium (RPMInorm) or RPMI1640 medium supplemented with 1 mM Ca2+ (RPMIsuppl). Human Vδ2+ T cells (live CD14- CD20- CD3+ γδTCRlow or Vγ2+ cells) were analysed for the expression of (a) CD69 and the production of (b) IL-2 and (c) IL-4. (d) Human MAIT cells (live CD14- CD20- CD3+ Vα7.2+ CD161+ cells) were analysed for the expression of CD69. (e) Human B cells (CD14- CD3- CD20+ cells) were analysed for the expression of CD69. (f) Human monocytes (CD3- CD20- CD14+ cells) were analysed for the production of IL-2. Summary graphs (left panels) and representative data (right panels) are shown for the cytokine data. Data were pooled from three (Vδ2+ T cells; n = 9) and four (MAIT cells, B cells, monocytes; n = 12) independent experiments with three samples each. (JPG) [file pone.0282037.s005.jpg]

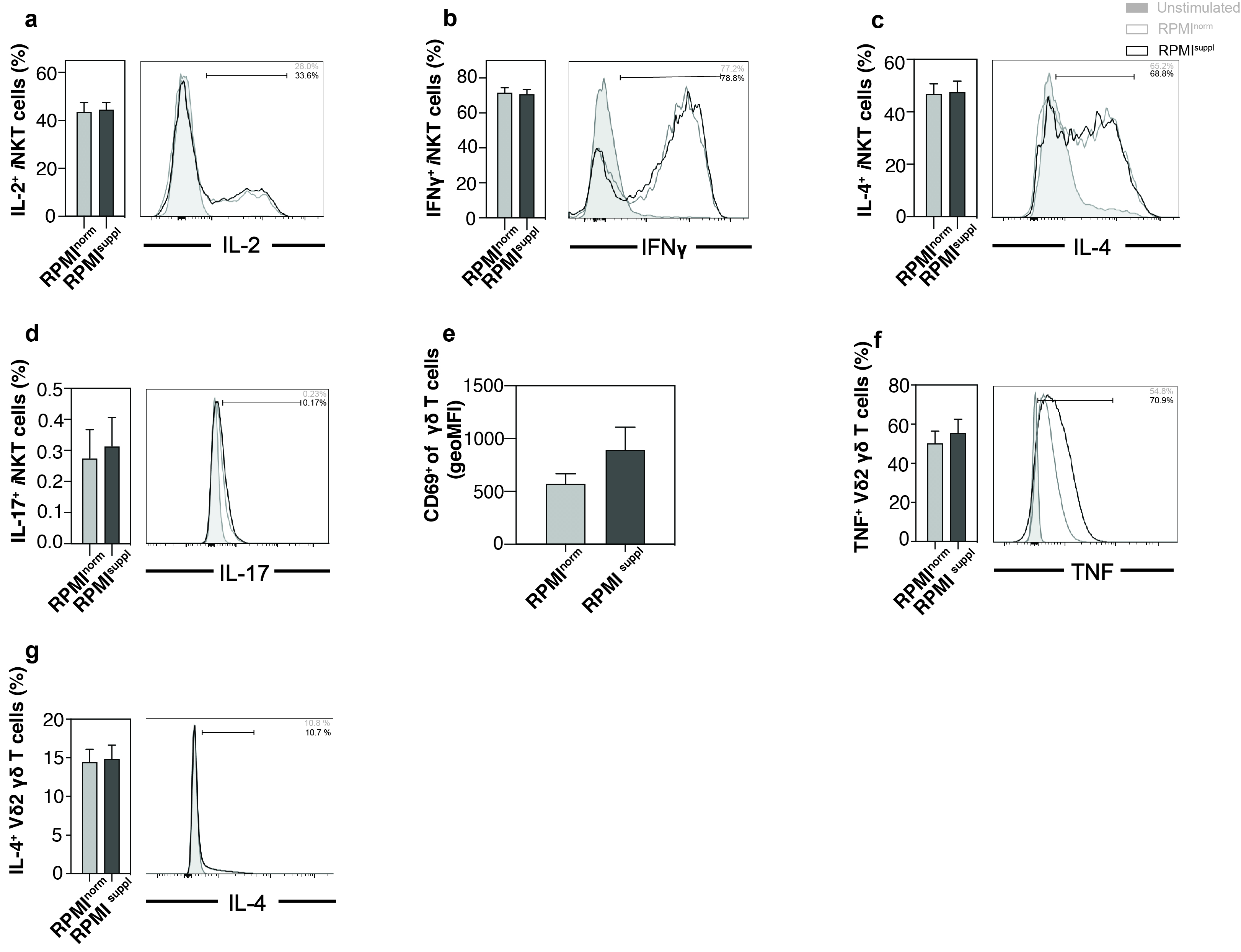

Supplement: S6 Fig — iNKT cells were expanded ex vivo in the presence of αGalCer. Vγ2+ T cells were expanded in vitro in the presence of Zoledronic acid. The expanded cells were stimulated for 4 h with 25 ng/ml PMA and 1 μg/ml ionomycin in either normal RPMI1640 medium (RPMInorm) or RPMI1640 medium supplemented with 1 mM Ca2+ (RPMIsuppl). Vα24i NKT cells (live CD14- CD20- CD3+ 6B11+ cells) were analysed for the production of (a) IL-2, (b) IFNγ, (c) IL-4, and (d) IL-17 were measured by ICCS. Human Vδ2+ T cells (live CD14- CD20- CD3+ Vγ2+ cells) were analysed for the expression of (e) CD69 and the production of (f) TNF, (g) IL-4. Summary graphs (left panels) and representative data (right panels) from gated iNKT cells and Vγ2+ T cells are shown, respectively. Data were pooled from four and three independent experiments with three samples each for iNKT cells (n = 12) and Vγ2+ T cells (n = 9), respectively. (JPG) [file pone.0282037.s006.jpg]

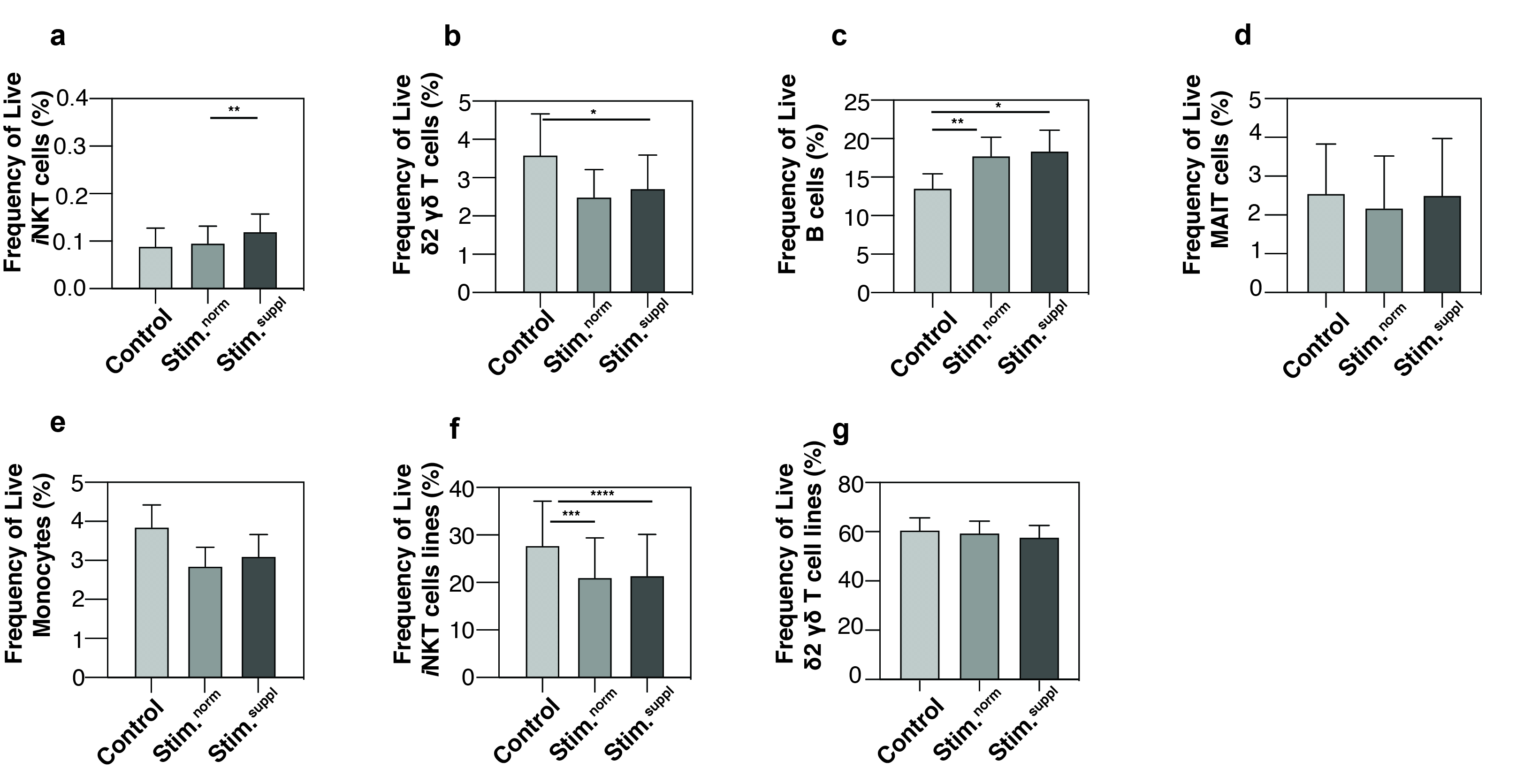

Supplement: S7 Fig — PBMCs were isolated from residual leukocyte units of healthy donors and were stimulated either directly (a-d) or after in vitro expansion of indicated cells (e, f). The cells were stimulated for 4 h with 25 ng/ml PMA and 1 μg/ml ionomycin in either normal RPMI1640 medium (RPMInorm) or RPMI1640 medium supplemented with 1 mM Ca2+ (RPMIsuppl). Primary (a) iNKT cells, (b) Vδ2+ T cells, (c) B cells; and (d) MAIT cells, (e) monocytes, or in vitro expanded (f) iNKT cells and (g) Vδ2+ T cells were stained and analysed by flow cytometry. The bar graphs show the relative percentages of cells positive for LIVE/DEAD Fixable Blue Dead Cell Stain, indicating dead cells. Data were pooled from three (a-b, g) or four (c—f) independent experiment with three samples each (n = 9–12). (JPG) [file pone.0282037.s007.jpg]

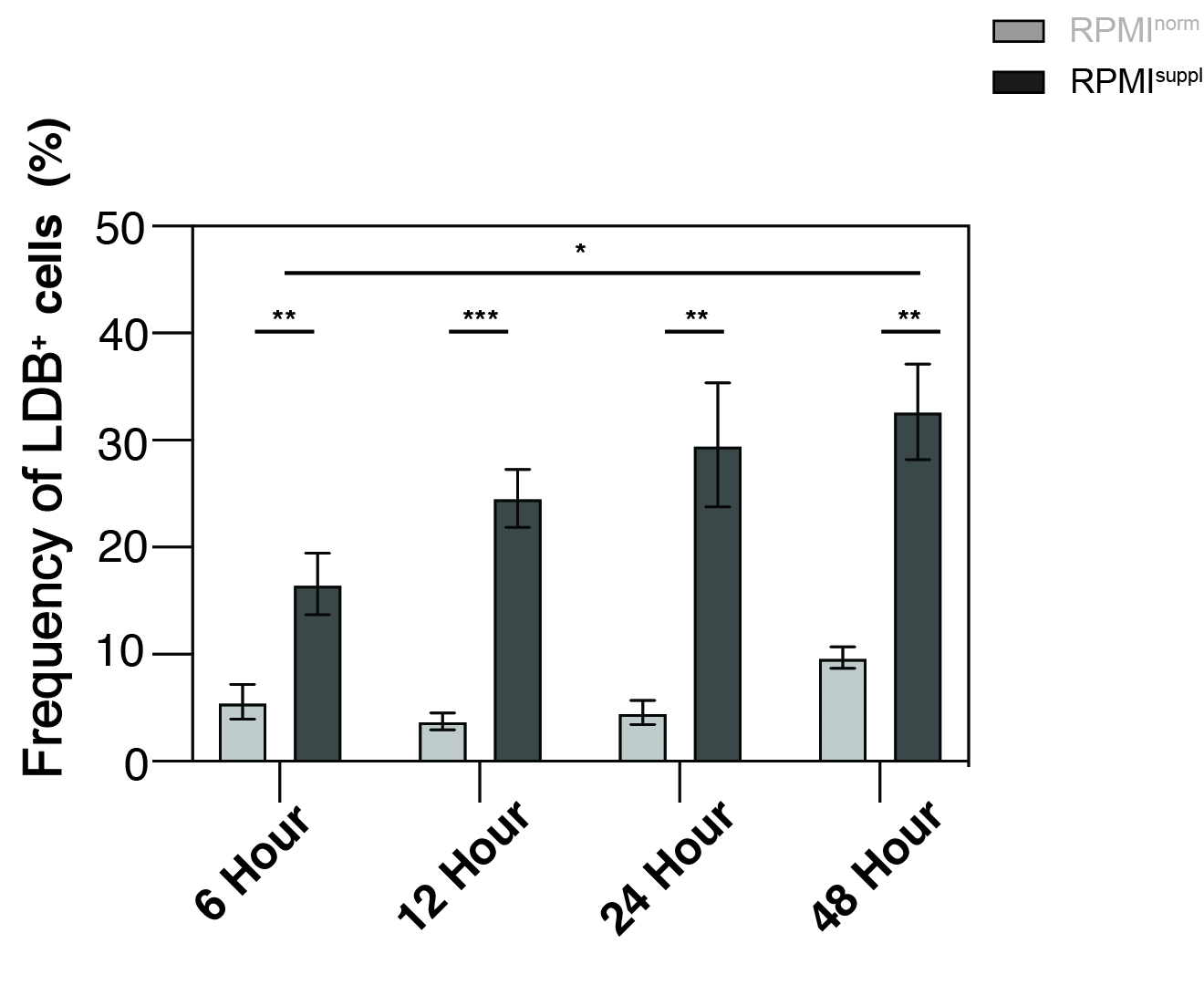

Supplement: S8 Fig — Primary human monocyte-derived macrophages were cultured for 6, 12, 24, or 48 hours in either normal RPMI1640 medium (RPMInorm) or RPMI1640 medium supplemented with 1 mM Ca2+ (RPMIsuppl). The bar graphs show the relative percentages of cells positive for LIVE/DEAD Fixable Blue Dead Cell Stain (LDB+ cells), indicating dead cells. Data shown are means ± SEM of biological replicates of six donors, pooled from two independent experiments with similar results. (JPG) [file pone.0282037.s008.jpg]

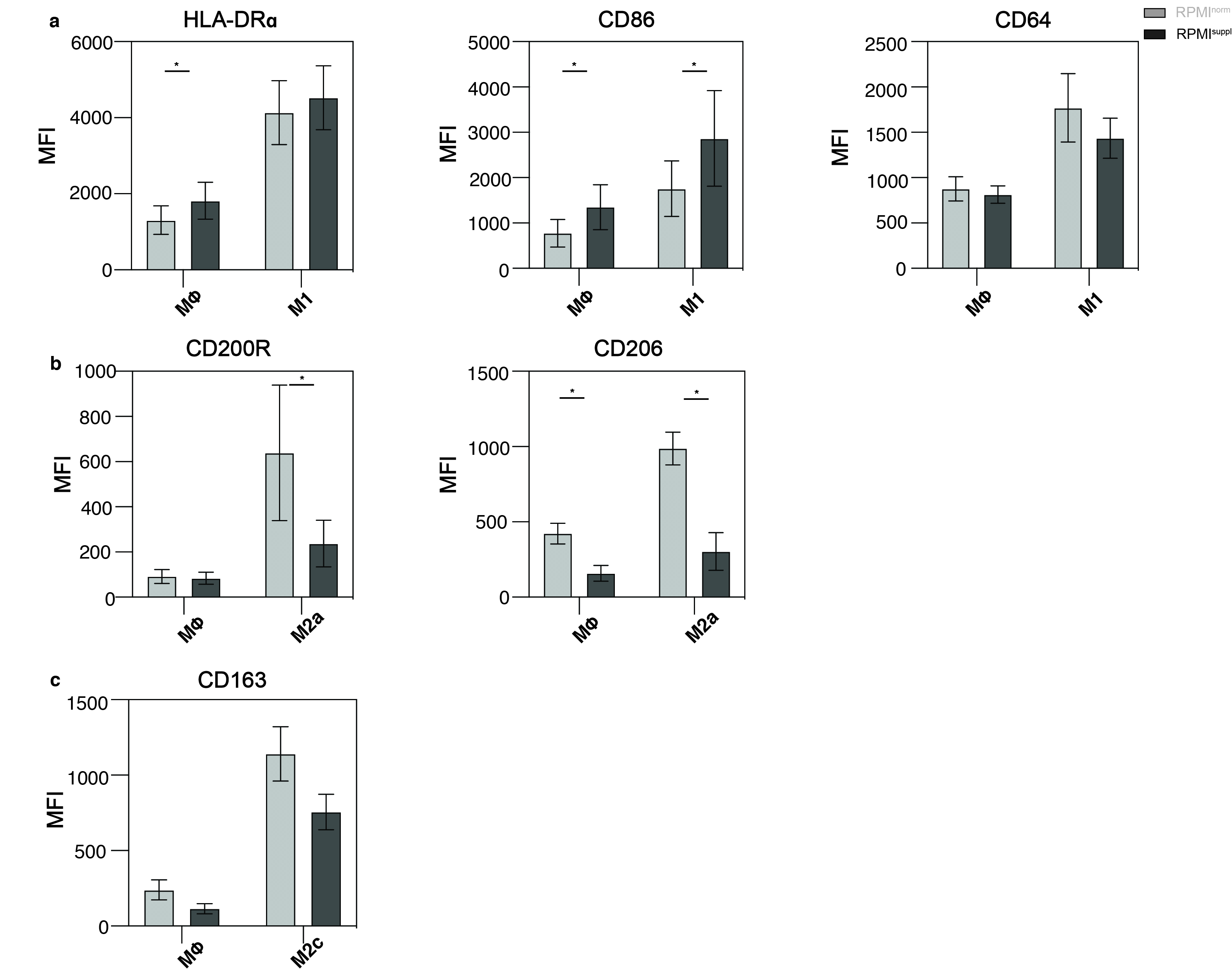

Supplement: S9 Fig — Primary human monocyte-derived macrophages were cultured untreated (MØ) or polarized into M1 (100ng/ml LPS, 20 ng/ml IFNγ, 12 hours), M2a (20 ng/ml IL-4, 24 hours), or M2c (20ng/ml IL-10, 24 hours) macrophages in either normal RPMI1640 medium (RPMInorm) or RPMI1640 medium supplemented with 1 mM Ca2+ (RPMIsuppl). The surface expression (mean fluorescent intensity, MFI) of indicated (a) M1 markers (HLA-DR, CD86, CD64), (b) M2a markers (CD200R, CD206), and an (c) M2c marker (CD163) are shown. The data shown are means ± SEM of biological replicates of six donors, pooled from two independent experiments with similar results. (JPG) [file pone.0282037.s009.jpg]
