# Supplementary material for: The Ca2+ concentration impacts the cytokine production of mouse and human lymphoid cells and the polarization of human macrophages in vitro
Source: PLoS One. 2023 Feb 24;18(2):e0282037. doi: 10.1371/journal.pone.0282037 (PMC9956017; doi:10.1371/journal.pone.0282037)
Supplement: S1 Table — (PDF) [file pone.0282037.s010.pdf]

S1 Table: Antibodies used in this study					
**Mouses targets**					
**Marker**	**Clone**	**Conjugate**	**Vendor**	**Identifier**	**Dilution**
CD3ε	145-2C11	BUV395	BD Biosciences	cat#563565, RRID:AB_2738278	1:100
CD4	RM4-5	BV605	BioLegened	cat#100548, RRID:AB_2563054	1:200
CD8α	53-6.7	BV570	BioLegened	cat#100740, RRID:AB_2563055	1:100
CD44	IM7	V500	BD Biosciences	Cat# 560780, RRID:AB_1937316	1:200
CD45R	RA3-6B2	BV650	BD Biosciences	Cat# 563893, RRID:AB_2738471	1:200
γδ TCR	UC7-13D5	FITC	eBiosciences	cat#11-5811-85, RRID:AB_465257	1:200
IFNγ	XMG1.2	BV785	BioLegened	cat#505838, RRID:AB_2629667	1:400
IL-2	JES5-16E3	PE-CF594	BD Biosciences	cat#562483, RRID:AB_11154219	1:800
IL-4	11B11	PE-Cy7	BioLegened	cat#504118, RRID:AB_10898116	1:200
IL-10	JES5-16E3	APC	BioLegened	cat#505010,RRID:AB_315364	1:200
IL-13	eBio13A	PerCP-eF710	eBiosciences	cat#46-7133-82, RRID:AB_11218496	1:400
IL-17A	TC11-18H10.1	APC-Cy7	BioLegened	Cat# 506940, RRID:AB_2565781	1:800
**Human targets**					
**Marker**	**Clone**	**Conjugate**	**Vendor**	**Identifier**	**Dilution**
CD3	UCHT1	BUV395	BD Biosciences	cat#563546,RRID:AB_2744387	1:400
CD14	M5E2	V500	BD Biosciences	cat#561391, RRID:AB_10611856	1:200
CD20	2H7	BV570	BioLegened	cat#302332, RRID:AB_2563805	1:200
CD64	10.1	PerCP-Cy5.5	BioLegened	cat#305024, RRID:AB_2561586	1:200
CD69	FN50	AF700	BioLegened	cat#310921, RRID:AB_493774	1:100
CD86	IT2.2	BV605	BioLegened	cat#305430, RRID:AB_2563824	1:400
CD161	HP-3G10	BV421	BioLegened	cat#339914, RRID:AB_2561421	1:40
CD161	HP-3G10	Pacific Blue	BioLegened	cat#339926, RRID:AB_2563960	1:50
CD161	HP-3G10	PerCP-Cy5.5	BioLegened	cat#339908, RRID:AB_2132862	1:100
CD163	GHI/61	PE-Cy7	BioLegened	cat#333614, RRID:AB_2562641	1:200
CD163	GHI/61	AF647	eBiosciences	cat#562669, RRID:AB_2737710	1:200
CD200R	OX-108	PE-Dazzle594	BioLegened	cat#329310, RRID:AB_2565526	1:400
CD206	15-2	AF700	BioLegened	cat#321132, RRID:AB_2616869	1:400
CXCL10	33036	AF647	R&D Systems	cat#IC266R	1:100
HLA-DR	L243	APC-Cy7	BioLegened	cat#307618, RRID:AB_493586	1:400
IFNγ	4S.B3	BV785	BioLegened	cat#502541, RRID:AB_11219192	1:100
IL-2	MQ1-17H12	BV650	BioLegened	cat#500334, RRID:AB_2563878	1:100
IL-4	8D4-8	PE-Cy7	eBiosciences	cat#25-7049-82, RRID:AB_469676	1:50
IL-10	JES3-9D7	eF660	eBiosciences	cat#50-7108-42, RRID:AB_11149363	1:50
IL-17	64DEC17	APC-eF780	eBiosciences	cat#47-7179-42, RRID:AB_11043559	1:100
iVα24	6B11	BV711	BioLegened	cat#342922, RRID:AB_2572068	1:80
iVα24	6B11	BV785	BioLegened	cat#342932,RRID:AB_2814264	1:200
γδTCR (pan)	B1	BV510	BioLegened	cat#331220, RRID:AB_2564275	1:50
TGFβ1	TW4-2F8	PE	BioLegened	cat#349604, RRID:AB_10645520	1:50
TNF	Mab11	PerCP-Cy5.5	eBiosciences	cat#45-7349-73, RRID:AB_953664	1:100
TNF	Mab11	AF700	BioLegened	cat#502928, RRID:AB_2561315	1:200
Vδ2	B6	AF700	BioLegened	cat#331415, RRID:AB_2687084	1:800
Vα7.2	3C10	FITC	BioLegened	cat#351704, RRID:AB_10900975	1:40
Vα7.2	3C10	BV711	BioLegened	cat#351732, RRID:AB_2629680	1:80
