# Supplementary material for: The Ca2+ concentration impacts the cytokine production of mouse and human lymphoid cells and the polarization of human macrophages in vitro
Source: PLoS One. 2023 Feb 24;18(2):e0282037. doi: 10.1371/journal.pone.0282037 (PMC9956017; doi:10.1371/journal.pone.0282037)
Supplement: Attachment — Submitted filename: Response to Reviewers.pdf [file pone.0282037.s011.pdf]

Gerhard Wingender
Izmir Biomedicine and Genome Center (IBG)
Dokuz Eylul University Health Campus
35340 Balcova/Izmir
Turkey
Email: gerhard.wingender@ibg.edu.tr

11 October 2022

RESPONSE TO THE REWIEVER & EDITORS:

We thank the reviewer and editors for their time and efforts in their review, which helped us to improve the manuscript. Enclosed please find the revised version of the manuscript that takes into account the raised comments and suggestions, and that highlights the important changes in the text in yellow. Please find below our point-to-point reply to the issues raised.

Editors

1. Please ensure that your manuscript meets PLOS ONE's style requirements, including those for file naming.

Response: We followed PLOS ONE's guidelines to our best knowledge throughout.

2. To comply with PLOS ONE submissions requirements, in your Methods section, please provide additional information on the animal research and ensure you have included details on (1) methods of sacrifice, (2) methods of anesthesia and/or analgesia, and (3) efforts to alleviate suffering.

Response: We added the method of sacrifice of the mice (cervical dislocation) to the Material and Methods section. All mouse experiments were performed *ex vivo*, therefore, no anaesthesia was required. However, we added a statement that we followed 3R guidelines to the relevant section in the Material and Methods section.

3. We note that you have stated that you will provide repository information for your data at acceptance. Should your manuscript be accepted for publication, we will hold it until you provide the relevant accession numbers or DOIs necessary to access your data. If you wish to make changes to your Data Availability statement, please describe these changes in your cover letter and we will update your Data Availability statement to reflect the information you provide.

Response: We ticked the option "URLs/accession numbers/DOIs" during the online submission by mistake. No data related to this manuscript need to be deposited and, therefore, we removed the tick now during the resubmission.

4. We note that the grant information you provided in the 'Funding Information' and 'Financial Disclosure' sections do not match. When you resubmit, please ensure that you provide the correct grant numbers for the awards you received for your study in the 'Funding Information' section.

Response: Unfortunately, this point is not entirely clear to us. We found on the editorialmanager.com website, the 'Funding Information' section (tab 'Manuscript Data') but not the 'Financial Disclosure' section. All information provided in the 'Funding Information' section is complete and correct. No information on funding was initially added to the manuscript text as the PLOS ONE guidelines (https://journals.plos.org/plosone/s/submission-guidelines) explicitly state "*Do not include funding sources in the Acknowledgments or anywhere else in the manuscript file. Funding information should only be entered in the financial disclosure section of the submission system*". Upon inquiry with PLOS ONE (plosone@plos.org), we were informed that "*The Financial Disclosure written in your manuscript must match your Funding Information in the Manuscript Data Tab. Therefore, we encourage you to correct your Financial Disclosure in your manuscript*." Following this advice, we now added a 'Financial disclosure' section to the main text.

Reviewer #1

1. If the author shift the word 'in vitro' to the end of the sentence the title sounds more familiar.

Response: We thank the reviewer for this suggestion and the title was changed accordingly.

2. In the method section author wrote that 'Antibodies were purchased 357 from either BioLegend (USA), BD Biosciences (USA), or eBiosciences (USA).' However, there is an international format for writing chemical reagents in a scientific paper. The author needs to be specific with company name by particular reagent. Furthermore, the author is suggested to recheck all manuscript to follow the same rule while mention company manes.

Response: We thank the reviewer to bring this ambiguity to our attention. We double-checked that Material & Method section and belief to have stated now the specific vendor for every reagent mentioned, including for the utilized antibodies.

3. The 'Cell preparation' section in Methods seeking brief explanation of procedures of cell isolations.

Response: We extended the information provided under 'Cell Preparation' to make the paragraph self-explanatory without the need to revert to previous publications.

4. The author used 1 mM CaCl2 for stimulating cells in vitro. What is the rationale of using this single concentration? Is this stimulation is dose independent?

Response: It was shown previously (Zimmermann *et al.* 2015, PMID: 25545753) that supplementing RPMI media with 1 mM of CaCl2 is required to obtain the maximal cytokine production by stimulated conventional $CD4^+$ T cells. The goal of our study

was to clarify whether this observation would also be relevant for unconventional T cells (*i*NKT cells, MAIT cells, $\gamma\delta$ T cells) and human macrophages (as an example of myeloid cells). Therefore, we think that Ca2^{+}-titration experiments would not be in line with our aim and would distract from our main finding.

5. Under the section 'Human macrophage polarization', the author wrote that 'The incubation times are specified in the text.' However, they are suggested to write it here. There is no problem if they want to keep it in the text as an addition.

Response: We thank the reviewer for bringing this omission to our attention. We added now the range of incubation times to the Material & Method section.

6. The authors are strongly suggested to add positive and negative controls in their experiment.

Response: We agree with the reviewer that negative and positive controls are essential for all experiments. All our experiments included an untreated (e.g. unstimulated) control (i.e. negative control). Our positive control was the cell stimulation with PMA/ionomycin (lymphoid cells, monocytes), LPS/IFNγ (M1), IL-4 (M2a), or IL-10 (M2c) in standard RPMI medium. Our experimental groups were the stimulation with Ca2^{+}-supplemented RPMI medium. Therefore, all our experiments were performed with the relevant negative and positive controls.

We believe that we have fully addressed all concerns raised and hope that our revised manuscript is now suitable for publication in *PLOS ONE*.

Please do not hesitate to contact me if you had any questions.

Yours sincerely,

G. Wingender

Gerhard Wingender
