# Supplementary material for: The Ca2+ concentration impacts the cytokine production of mouse and human lymphoid cells and the polarization of human macrophages in vitro
Source: PLoS One. 2023 Feb 24;18(2):e0282037. doi: 10.1371/journal.pone.0282037 (PMC9956017; doi:10.1371/journal.pone.0282037)
Supplement: Attachment — Submitted filename: Point-to-point reply_R2.pdf [file pone.0282037.s012.pdf]

Gerhard Wingender
Izmir Biomedicine and Genome Center (IBG)
Dokuz Eylul University Health Campus
35340 Balcova/Izmir
Turkey
Email: gerhard.wingender@ibg.edu.tr

23 December 2022

RESPONSE TO THE REWIEVER & EDITORS:

We thank the reviewers and editors for their time and efforts in their review, which helped us to improve the manuscript. Enclosed please find the revised version of the manuscript that takes into account the raised comments and suggestions, and that highlights the important changes in the text in yellow. Please find below our point-to-point reply to the issues raised.

Reviewer #1

The author answered that 'It was shown previously (Zimmermann et al. 2015, PMID: 25545753) that supplementing RPMI media with 1 mM of CaCl2 is required to obtain the maximal cytokine production by stimulated conventional CD4+ T cells.' They are suggested to add this explanation in their manuscript in the most suitable place.

Response: We thank the reviewer for this suggestion and we highlighted this point better now in the introduction.

Reviewer #2

The manuscript highlights an observation known for decades but not addressed in detail.

Response: That calcium in the medium is require for *in vitro* responses by immune cells is indeed know for decades, but this is not the topic of our study. That the calcium concentration in the commonly used RPMI1640 medium is insufficient for maximal cytokine responses of conventional T cells was first reported in 2015. We now extent this finding to other, unconventional T cells (*i*NKT cells, MAIT cells, γδ T cells) and to macrophages as an example of myeloid cells. We do believe that our data are of significant interest to researchers utilizing *in vitro* experiments to study unconventional T cells and macrophages.

1.) The authors use standard isolation procedures to isolate T cell subsets and they polarize monocytes to macrophages M1/M2 but do not show the success of their manipulation. Pheno/genotypic markers need to be used to precisely characterize the invariant NK-T cells, mucosal-associated invariant T cells, γδ T cells after isolation/stimulation. The same is true for monocyte-macrophage differentiation.

Response: To illustrate our gating and the purity of the analysed cell populations, we now included a new supplemental figure (S1 Fig).

2.) I have a lot of issues open concerning the description of the methods and materials used.
a. For human samples an approval number of the Ethic committee is provided but this is not the case for animals.

Response: This information was already provided in the manuscript, under the section 'Mice': "All mouse experiments were performed with prior approval by the institutional ethic committee ('Ethical Committee on Animal Experimentation' of the Izmir Biomedicine and Genome Center, approval number: 19/2016) in accordance with national laws and policies."

b. ARRIVE guidelines are cited with no reference

Response: We thank the reviewer for pointing out this inadvertent omission. We added the reference in the revised version of the manuscript.

c. All antibodies used were given with a clone identification but without the given concentration and conjugation with either Brilliant Ultra Violet 395, Pacific Blue, Violet 500, Brilliant Violet 570,358 Brilliant Violet 605, Brilliant Violet 650, Brilliant Violet 711, Brilliant Violet 785, FITC, PerCP-359 Cy5.5, PerCP-eF710, PE, PE-CF594, PE-Dazzle594, PECy7, APC, AF647, eF660, AF700, 360 APC-Cy7, or APC-eF780 and therefor it is hard to follow.

Response: To further clarify this point, we added a new supplemental table showing the clone, conjugate, source, ID, and dilution for each antibody used in this study.

d. No concentration is given on the blocking mAbs.

Response: We assume the reviewer refers to the reagents used to block the Fc receptors. We thank the reviewer for pointing out this inadvertent omission. We added now the clarification "… according to the manufacturers' recommendations"

e. What kind of Flow Cytometer is used and which program for calculation and graphical display?

Response: We thank the reviewer for pointing out this inadvertent omission. This information has been added now to the Material and Methods section.

f. When it comes to statistic paired tests are not appropriate and it is easy for the authors to provide unpaired alternative tests.

Response: The use of paired tests for the statistical analysis of the reported data (cell frequencies in percentages, geometric MFIs) is in line with the common procedure in the field. Furthermore, we previously verified internally that such data are normally distributed (D'Agostino - Pearson omnibus normality test after combining values from repetitive experiments (n>10, GraphPad Prism)), demonstrating in our opinion that a paired test is appropriate. However, should the reviewer outline for which experiment the usage of a paired test is inappropriate and why, we are happy to revaluate our analysis.

3.) The reviewer has the impression that the authors show too many Figures 1-7 and additional half-a-dozen Figures in the Supplements. I would recommend to extract the important issues and combine Figures, describe the irrelevant in the results and add the necessary controls of T cell and macrophage subsets in the Supplements.

Response: We understand the concern of the reviewer; however, we think that combining figures or removing parts of them would reduce the clarity or required information content, respectively.

Minor comments:

1.) When providing information on materials used, the authors should provide more information. BioLegend should read BioLegend® Inc., San Diego, CA for the first time and thereafter BioLegend every second/third time. I 'know' this is work.

Response: We did not see the usage of ® in other recent PLOS One publications and, similar, the location of the company headquarters appears not to be a journal requirement. However, we included now the requested information on the company headquarters in the revised manuscript.

2.) Also the English language needs to be improved for clarity.

Response: If the reviewer refers to the English in which the manuscript is written, then we are surprised to see this comment, as we are confident that the understandability and clarity of the English utilized throughout meets PLOS One standards, not alone as three papers from the laboratory were recently (2021, 2022) published in PLOS One. However, should the reviewer point out specific issues, we would be happy to improve the text were necessary.

We believe that we have fully addressed all concerns raised and hope that our revised manuscript is now suitable for publication in *PLOS ONE*.

Please do not hesitate to contact me if you had any questions.

Yours sincerely,

G. Wingender

Gerhard Wingender
